# Digital Self-Interference Canceler with Joint Channel Estimator for Simultaneous Transmit and Receive System

**DOI:** 10.3390/s24082449

**Published:** 2024-04-11

**Authors:** Shiyu Song, Yanqun Tang, Xianjie Lu, Yu Zhou, Xizhang Wei, Zhengpeng Wang, Songhu Ge

**Affiliations:** 1 School of Electronics and Communication Engineering, Sun Yat-sen University, Shenzhen 518107, China; songshy7@mail2.sysu.edu.cn (S.S.); luxj25@mail2.sysu.edu.cn (X.L.); zhouy633@mail2.sysu.edu.cn (Y.Z.); weixzh7@mail.sysu.edu.cn (X.W.); wangzhp26@mail2.sysu.edu.cn (Z.W.); 2 National Key Laboratory of Electromagnetic Energy, Naval University of Engineering, Wuhan 430033, China; gesonghu@126.com

**Keywords:** simultaneous transmit and receive, self-interference cancellation, digital cancellation, adaptive filtering, joint channel estimator

## Abstract

Simultaneous transmit and receive wireless communications have been highlighted for their potential to double the spectral efficiency. However, it is necessary to mitigate self-interference (SI). Considering both the SI channel and remote transmission (RT) channel need to be estimated before equalizing the received signal, we propose two adaptive algorithms for linear and nonlinear self-interference cancellation (SIC), based on a multi-layered joint channel estimator structure. The proposed algorithms estimate the RT channel while performing SIC, and the multi-layered structure ensures improved performance across various interference-to-signal ratios. The M-estimate function enhances the robustness of the algorithm, allowing it to converge even when affected by impulsive noise. For nonlinear SIC, this paper introduces an adaptive algorithm based on generalized Hammerstein polynomial basis functions. The simulation results indicate that this approach achieves a better convergence speed and normalized mean squared difference compared to existing SIC methods, leading to a lower system bit error rate.

## 1. Introduction

Full duplex (FD) relay technology enables a communication system to simultaneously transmit and receive signals on the same frequency through a relay node [1]. Inspired by this technology, simultaneous transmit and receive (STAR) technology, also referred to as in-band full-duplex (IBFD), has emerged as a novel innovation within the field. It aims to allow communication users to directly transmit and receive signals at the same carrier frequency, facilitating an even more efficient use of spectral resources. Moreover, STAR technology can be synergistically combined with other advanced technologies that enhance spectral utilization, such as reconfigurable intelligent surfaces [2], millimeter-wave technology [3], and multiple access techniques [4]. As a result, STAR technology has arisen as a promising approach to increase data throughput and solve the problem of scarce spectrum resources, which can be applied in the field of wireless communications, radar, satellites, and unmanned aerial vehicles [5,6].

**Figure 1:**
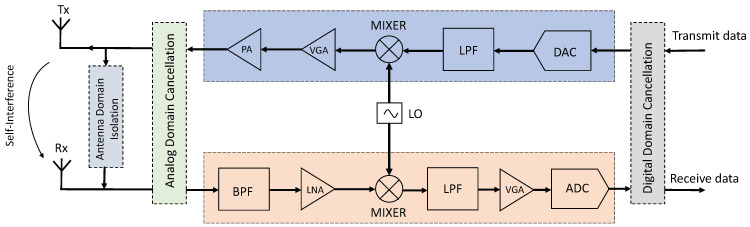
A block diagram illustrating the STAR system and various forms of SIC.

However, the self-interference (SI) signal, which is generated from the near end, can be 100–120 dB stronger than the weak received signal, thus making the receiver inoperable [7]. Therefore, self-interference cancellation (SIC) technology is key to the implementation of STAR communications. At present, the SI signal can be eliminated in several stages and various domains, as shown in Figure 1. Typically, SIC is achieved through the cascaded antenna domain [8], analog domain [9], and digital domain [10,11,12,13,14,15]. The antenna domain SIC is passive in nature, and mainly uses antenna isolation or related beamforming algorithms to prevent RF reception path blockage. In the analog domain, directly coupled interference suppression and digital-assisted analog interference-suppression methods are commonly used at the receiver low-noise amplifier (LNA) input before the analog-to-digital conversion. The last stage is digital SIC, which aims to eliminate the remaining SI from the received signal by reconstructing the SI signal in the receiving link and reducing its power below the noise floor. These steps have been applied in some prototypes and have demonstrated the potential of STAR communications [7,16]. The remainder of this paper focuses on digital SIC algorithm design and both the linear and nonlinear SIC algorithms are evaluated through extensive simulations, demonstrating their effectiveness in improving the performance of STAR communication systems.

Digital SIC has attracted significant attention in recent years to address the SI problem in STAR communication systems. Nonetheless, the practical application of digital SIC still faces many challenges. First of all, the observed SI signal exhibits significant frequency and time selectivity due to reflections within the device and its surrounding environment, which demonstrate temporal variations. This can be regarded as a classic system identification problem, which is typically solved through adaptive finite impulse response (FIR) filters. Secondly, in practical communication environments, there are often additional sources of noise, such as electromagnetic interference, lightning noise, radar signals, and other human-made disturbances. These types of noise typically exhibit strong pulse-like characteristics within very short time durations, which can lead to performance degradation of various filtering algorithms based on the assumption of Gaussian noise. Furthermore, the significantly higher power of the SI signal compared to the desired signal at the receiver implies that even minor distortions can significantly degrade the signal of interest. These hardware impairments, such as phase noise, in-phase/quadrature (I/Q) imbalance, and power amplifier (PA) and baseband nonlinearity, among others, with PA nonlinearity are especially harmful to the digital canceler, making digital SIC insufficient.

Digital cancellation algorithms accounting for impulsive noise have been proposed in earlier research [10,17]. The work in [11,12] focused on linear SIC by employing the least mean square (LMS) adaptive filter. In addition, different linear SIC methods were compared in [13]. To compensate for nonlinear PA distortion, the authors in [18] proposed novel predistorters and their parameter extraction algorithms. The proposed self-adaptive nonlinear digital cancelers in [14,15] both utilize a novel orthogonalization procedure for nonlinear basis functions, together with low-cost LMS-based parameter learning. Both PA nonlinearity and I/Q imbalances were considered in [19,20], and more impairments, including local oscillator leakage and baseband nonlinearity, were analyzed and measured on the Universal Software Radio Peripheral in [7]. Some studies have also proposed various frequency domain SIC methods [21,22]. In [21], a frequency domain SI canceler based on the parallel Hammerstein (PH) model for an IBFD orthogonal frequency division multiplexing System was proposed, while Ref. [22] utilized a successive cancellation cascaded structure as a replacement for the adaptive filter, effectively reducing the computational load of SIC processing and simplifying the procedure. Most published works are based on the scenario where only the own transmitter (TX) signal is known, without considering the remote transmission (RT) signal. When the RT sends a signal for channel estimation, it can be assumed to be known, and additional prior information will further enhance the performance of SIC [23].

In this paper, a robust multi-layered M-estimate total least mean square (m-MTLS) canceler is proposed to enhance the performance in scenarios where both the data matrix and the observation signal are also affected by impulsive noise. The method exhibits significant robustness against impulse noise, managing to maintain a low convergence error even in the presence of impulse noise. When both the PA nonlinearities of the local transmitter and the training signal from RT are taken into account, we introduce a novel algorithm for nonlinear digital SIC based on a set of generalized Hammerstein polynomials (HPs) [15], by estimating the SI channel and the RT channel at the same time. This method of joint estimation can more accurately estimate the remote channel while reducing the residual SI signal, compared with existing SIC methods. The simulation results show that this method exhibits a faster convergence rate and demonstrably enhances system performance as the signal-to-noise ratio (SNR) increases.

The rest of this paper is organized as follows. In Section 2, the basic STAR system and linear SI signal modeling are first described. Then, considering the nonlinearity introduced by the PA, the SI model is extended to a nonlinear model, followed by the introduction of a joint estimator that can simultaneously estimate both SI and RT channels. Section 3 proposes two new algorithms for linear and nonlinear SIC based on adaptive FIR, respectively. The linear SIC also enhances the robustness of the algorithm under impulsive noise interference. The simulation results are reported in Section 4. Finally, Section 5 concludes the paper.

In the following, continuous and discrete time signals are expressed in italic lowercase, while vectors are denoted by bold lowercase symbols. The acronyms used throughout the paper are also summarized in Table 1.

## 2. System Model

**Figure 2:**
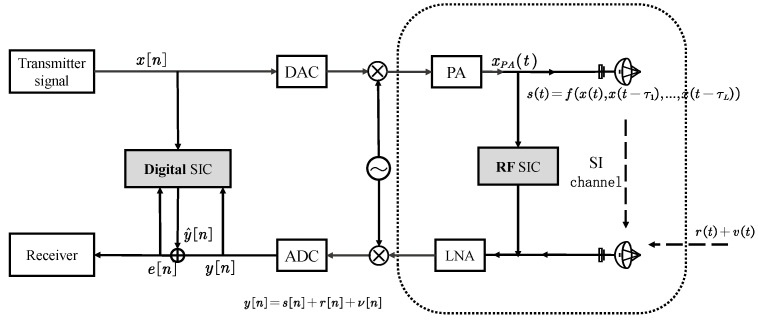
The signals propagating at the different stages of the analyzed STAR system.

The structure of the analyzed STAR system is presented in Figure 2, with signals propagating at the different stages. We denote the baseband transmission at time slot *n* as x[n], which is perfectly known. In this analysis, the RF components are assumed to be ideal and the nonlinear model will be discussed later. The received signal, after being processed by the LNA and and subjected to SIC in the analog domain, is converted into a baseband signal by the ADC at time slot *n* and can be written as

(1) $$\begin{array}{cc} y[n] & =r[n]+f(x[n],x[n-1],\ldots x[n-L+1])+v[n]\\ \; & =r[n]+s[n]+v[n], \end{array}$$

where r[n] and v[n] denote the desired signal from the remote source and noise at time instant *n*, respectively. The received signal is assumed to be generated by a function f(·) with *L* previous transmitted signals. Furthermore, the SI signal from the local TX, s[n], can be represented by a linear model as

(2) $$\begin{array}{cc} s[n] & =f(x[n],x[n-1],\ldots ,x[n-L+1])\\ \; & =w^{T}\left[n\right]x_{L}\left[n\right], \end{array}$$

where $w\in R^{N\times 1}$ denotes the SI channel and $x_{L}\left[n\right]=\left[x\left[n\right],x\left[n-1\right],\ldots ,x\left[n+L-1\right]\right]$.

When the nonlinear hardware impairments are taken into account, one particularly important imperfection is PA nonlinearity; thus, we assume that the transmitted signal is only distorted by high-order harmonics of the PA, excluding the effects of I/Q imbalance or phase noise. The PAs exhibit nonlinearity when power-efficient operation is sought, and it is commonly modeled with the well-known PH model [14,19,22,24]. With an input vector $x_{L}\left[n\right]$, the output of a *P*-th order model can be expressed as

(3) $$\begin{array}{cc} x_{\mathrm{PA}}\left(t\right) & =\sum_{\begin{array}{c} p=1\\ p\;\mathrm{odd}\; \end{array}}^{\frac{P+1}{2}}h_{p,\mathrm{PA}}\left(t\right)*\phi_{p}\left(x\left(t\right)\right), \end{array}$$

where *P* is the highest nonlinearity order of the model, $h_{p,PA}\left(t\right)$ represents the response, and $\phi_{p}\left(x\left(t\right)\right)={\left|x(t)\right|}^{2p}x\left(t\right)$ is the *p*th-order basis function. The symbol ∗ represents the convolution operation. We assume that the PA memory length is $L_{1}$ and the length of the SI channel is $L_{2}$. Then, the observed SI signal passes through the SI channel and can be rewritten as

(4) $$\begin{array}{cc} s[n] & =\sum_{\begin{array}{c} p=1\\ p\;\mathrm{odd}\; \end{array}}^{\frac{P+1}{2}}\sum_{l=0}^{L-1}w_{p}\left[l\right]\phi_{p}\left(x\left[n-l\right]\right), \end{array}$$

where $w_{p}$ is the response weight of the entire channel, with a length of $L=L_{1}+L_{2}-1$.

Building on the analysis presented earlier, for the purpose of mitigating residual SI within the digital domain, it is imperative to acquire the optimal estimate of the SI channel response, denoted as $\hat{w}\left[n\right]$, corresponding to w[n]. This involves subtracting the reconstructed SI signal from y[n]. Followed by the SIC process, the remainder of the signal is presumed to embody the RT signal. Subsequent stages may include RT channel estimation and equalization, facilitating the detection of data symbols embedded within the RT signal.

From the SI model mentioned in (2) and (4), it is evident that the performance of digital SIC is fundamentally contingent upon the accuracy of the channel response coefficient estimation. Moreover, the power of the residual SI signal remains high after RF cancellation. when the interference-to-signal ratio (ISR) is high, even minimal residuals can severely compromise the separation of the RT signal. Additionally, at higher SNRs, noise ceases to be the predominant factor impacting the estimation of the SI signal. Under such circumstances, the primary source of error in SIC emanates from the RT signal itself. Consequently, in scenarios where the RT signal is known, it becomes essential to employ a joint channel estimator to enhance the precision of both SI and RT signal separations, thereby mitigating the aforementioned challenges. We assume that the original RT signal, i[n], is known; then, (1) and (2) can be rewritten in [23] as

(5) $$\begin{array}{c} y\left[n\right]=w^{T}\left[n\right]x\left[n\right]+h^{T}\left[n\right]i\left[n\right]+v\left[n\right], \end{array}$$

where h[n] denotes the RT channel coefficient, and the interest signal, r[n] is represented as $h^{T}\left[n\right]i\left[n\right]$.

By merging two channels into a joint channel, according to (5), we can obtain the following expression:

(6) $$\begin{array}{c} y\left[n\right]=c^{T}\left[n\right]u\left[n\right]+v\left[n\right], \end{array}$$

where $c^{T}\left[n\right]=\left[w^{T}\left[n\right],h^{T}\left[n\right]\right]$ combines the coefficients of both the SI and RT channels, and $u\left[n\right]={\left[x^{T}\left[n\right],i^{T}\left[n\right]\right]}^{T}$ is the new input signal.

In (5), the estimation of SI is influenced by both $r\left[n\right]=h^{T}\left[n\right]i\left[n\right]$ and v[n]. However, by combining the SI and RT channel as a new estimation parameter, only the noise v[n] impacts this process. The benefits of this joint channel estimation approach will be detailed in the simulations presented in Section 4.

## 3. Proposed Digital Canceler

**Figure 3:**
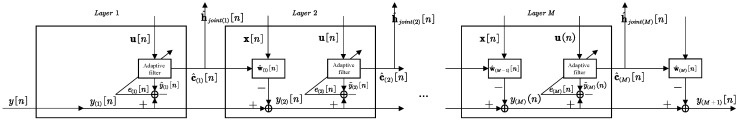
The multi-layered structure of the joint channel estimator.

When considering the SI and RT channels, it is important to acknowledge that they will vary over time, as the environmental reflection paths are also time-variant. Therefore, we opted for adaptive filtering algorithms and incorporated a multi-layered structure, as shown in Figure 3, to enhance the RT estimation performance of the joint estimator.

As depicted in Figure 3, the estimation of joint channel coefficients $\hat{c}\left[n\right]$ in the first layer is given by ${\hat{c}}_{\left(1\right)}^{T}\left[n\right]=\left[{\hat{w}}_{\left(1\right)}^{T}\left[n\right],{\hat{h}}_{\left(1\right)}^{T}\left[n\right]\right]$; then, the residual signal $y_{\left(2\right)}\left[n\right]$, obtained by subtracting the reconstructed SI signal from y[n], serves as the input for the second layer. Furthermore, we can obtain

(7) $$\begin{array}{cc} y_{\left(2\right)}\left[n\right] & =c_{\left(1\right)}^{T}\left[n\right]u\left[n\right]-{\hat{w}}_{\left(1\right)}^{T}\left[n\right]x\left[n\right]+v\left[n\right]\\ \; & =c_{\left(2\right)}^{T}\left[n\right]u\left[n\right]+v\left[n\right]. \end{array}$$

This procedure is consistently implemented across successive layers of the filter. In the *m*-th layer, this process is articulated as

(8) $$\begin{array}{cc} y_{\left(m+1\right)}\left[n\right] & =y_{\left(m\right)}\left[n\right]-{\hat{w}}_{\left(m\right)}^{T}\left[n\right]x\left[n\right]+v\left[n\right]\\ \; & =c_{\left(m+1\right)}^{T}\left[n\right]u\left[n\right]+v\left[n\right] \end{array},$$

where $y_{\left(m\right)}\left[n\right]$ is the input of the *m*-th layer and ${\hat{w}}_{\left(m\right)}\left[n\right]$ is the estimation of the SI channel, and we have ${\hat{c}}_{\left(m\right)}^{T}\left[n\right]=\left[{\hat{w}}_{\left(m\right)}^{T}\left[n\right],{\hat{h}}_{\left(m\right)}^{T}\left[n\right]\right]$ as the estimation of the joint channel.

For each layer, while subtracting the residual from the received signal, an estimation of the RT channel ${\hat{h}}_{\left(m\right)}\left[n\right]$ is performed and the output of the last layer $y_{\left(M+1\right)}\left[n\right]$ is regarded as the interest signal $\hat{r}\left[n\right]$.

### 3.1. Robust m-MTLS Adaptive Algorithm

We consider a linear model where both the independent and dependent variables are subject to measurement errors, which can be illustrated by

(9) $$\left(y_{n}+v_{n}\right)={\left(x_{n}+u_{n}\right)}^{T}w,$$

where $w\in R^{L\times 1}$ denotes the system vector to be estimated, with $x_{n}\in R^{L\times 1}$ as the input vector and $y_{n}\in R$ as the output signal at time *n*. The noise vectors $u_{n}$, distributed as $N\left(0,\sigma_{i}^{2}I\right)\in R^{L\times 1}$, represent the input noise, and $v_{n}$, distributed as $N(0,\sigma_{o}^{2})\in R$, represent the output noise, where $\sigma_{i}^{2}$ and $\sigma_{o}^{2}$ are the variances in the input and output noise, respectively. Here, 0 indicates the zero vector and I the identity matrix.

Total least squares (TLS) considers errors in all variables, making it particularly useful for more accurate modeling and estimation when errors are present in both predictors and outcomes [25]. Its cost function can be formulated as

(10) $$\min_{w}J\left(w\right)=\frac{1}{N}\sum_{n=1}^{N}\frac{{\left({\tilde{y}}_{n}-w^{T}{\tilde{x}}_{n}\right)}^{2}}{{∥w∥}^{2}+\gamma },$$

where $\gamma \overset{\mathrm{def}}{=}\sigma_{o}^{2}/\sigma_{i}^{2}$ is a parameter to normalize the noise variances, ${\tilde{y}}_{n}=y_{n}+v_{n}$ and ${\tilde{x}}_{n}=x_{n}+u_{n}$.

Similar to other adaptive algorithms, the iterative formula for TLS based on stochastic gradient descent can be expressed as

(11) $$\begin{array}{cc} {\hat{w}}_{n+1} & ={\hat{w}}_{n}-\mu \hat{g}\left({\hat{w}}_{n}\right)\\ \; & ={\hat{w}}_{n}+\mu \alpha_{n}\left({\tilde{x}}_{n}+\alpha_{n}{\hat{w}}_{n}\right), \end{array}$$

where μ is the step-size parameter, and $\hat{g}$ is the instantaneous gradient of J(w), which can be represented as

(12) $$\begin{array}{cc} \; & \hat{g}\left(w\right)=\alpha_{n}\left({\tilde{x}}_{n}+\alpha_{n}w_{n}\right),\\ \; & \alpha_{n}\overset{\mathrm{def}}{=}\frac{{\tilde{y}}_{n}-{\tilde{x}}_{n}^{T}w_{n}}{{∥w_{n}∥}^{2}+\gamma }. \end{array}$$

To enhance the robustness of the algorithm when the received signal is subjected to impulsive noise, an M-estimate function can be utilized to improve TLS. This improved algorithm, referred to as the M-estimate total least mean square (MTLS) adaptive algorithm, has its cost function defined as

(13) $$\min_{w}J\left(w\right)=E\left[\frac{\rho (e_{n}^{2})}{{∥w∥}^{2}+\gamma }\right],$$

where ρ(·) is the M-estimate function, which is a real-valued even function, given by

(14) $$\rho \left(e_{n}\right)=\left\{\begin{array}{cc} e_{n}^{2}/2, & |e_{n}|<\xi \\ \xi^{2}/2, & |e_{n}|\geq \xi , \end{array}\right.$$

where $\xi =c_{1}\hat{\sigma_{e}}$ represents the threshold parameter, with $c_{1}$ set to 2.576, and $\hat{\sigma_{e}}$ can be calculated by

(15) $${\hat{\sigma }}_{e_{n}}^{2}=\lambda_{\sigma }{\hat{\sigma }}_{e_{n-1}}^{2}+c_{2}\left(1-\lambda_{\sigma }\right)\mathrm{med}\left(A_{e_{n}}\right),$$

where $A_{e}\left[n\right]=\left[e^{2}\left[n\right],e^{2}\left[n-1\right],\ldots ,e^{2}\left[n-N_{w}+1\right]\right]$, med(·) denotes the median operation, and the parameter $\lambda_{\sigma }$, indicative of the weighting factor, is typically selected within the range of 0.98 to 0.99. The constant $c_{2}=1.483\left(1+5/\left(N_{w}-1\right)\right)$, and $N_{w}$ represents the length of the window over which the estimation is performed.

The iterative Formula (11) can be rewritten as

(16) $$\hat{g}\left(w\right)=\left\{\begin{array}{cc} -\frac{\left({∥w∥}^{2}+\gamma \right)e_{n}{\tilde{x}}_{n}+e_{n}^{2}w}{{\left({∥w∥}^{2}+\gamma \right)}^{2}}, & |e_{n}|<\xi \\ 0, & |e_{n}|\geq \xi , \end{array}\right.$$

and

(17) $$\begin{array}{cc} {\hat{w}}_{n+1} & ={\hat{w}}_{n}-\mu \hat{g}\left({\hat{w}}_{n}\right). \end{array}$$

By integrating the MTLS adaptive algorithm with the multi-layered joint channel estimator discussed in Section 2, a robust linear SI canceler can be achieved [10].

### 3.2. Multi-Layered Generalized HP-Based Adaptive Algorithm

Nonlinear SIC can be regarded as a problem of nonlinear system identification. Before engaging in adaptive filtering, it is necessary to linearize the SI signal using the basis functions described in (4).

The nonlinear PA model first proposed in [15] employs a set of generalized HPs for its representation. The *p*-th order nonlinear basis function in Equation (3) is defined as

(18) $$\begin{array}{c} \phi_{p}\left(x\left(t\right);c_{p}\right)=\sum_{k=0}^{p-1}c_{p,k}{\left|x(t)\right|}^{2k}x\left(t\right) \end{array},$$

which can be rewritten in the digital domain as

(19) $$\begin{array}{c} \phi_{p}\left(x\left[n\right];c_{p}\right)=\sum_{k=0}^{p-1}c_{p,k}{\left|x\left[n\right]\right|}^{2k}x\left[n\right] \end{array},$$

where $c_{p}={\left[c_{p,0},\ldots c_{p,p-1}\right]}^{T}$ represents the polynomial coefficients of the *p*-th basis function.

When the polynomial coefficients satisfy $c_{p,p-1}=1$ and $c_{p,k}=0,\forall k\neq p-1$, the basis function simplifies to the standard HP basis function, i.e., $\phi_{p}\left(x\left[n\right]\right)={\left|x\left[n\right]\right|}^{p-1}x\left[n\right]$. Then, the SI signal s[n] can be represented by the aforementioned generalized HP as

(20) $$\begin{array}{cc} s\left[n\right] & =\sum_{p=1}^{\frac{P+1}{2}}\sum_{l=0}^{L-1}w_{p}\left[l\right]\phi_{p}\left(x\left[n-l\right];c_{p}\right)\\ \; & =\sum_{p=1}^{\frac{P+1}{2}}\sum_{l=0}^{L-1}w_{p}\left[l\right]\left(\sum_{k=0}^{p-1}c_{p,k}{\left|x\left[n-l\right]\right|}^{2k}x\left[n-l\right]\right). \end{array}$$

To enhance the convergence speed of the adaptive filtering algorithm, it is also necessary to determine the coefficients $c_{p}={\left[c_{p,0},\ldots c_{p,p-1}\right]}^{T}$ of the generalized HP basis functions to ensure their orthogonality. A set of orthonormal basis functions is defined as $\left\{\phi_{1}\left(x_{L}\left[n\right];c_{1}\right),\ldots ,\phi_{\frac{P+1}{2}}\left(x_{L}\left[n\right];c_{\frac{P+1}{2}}\right)\right\}$, which satisfies the following orthogonality condition:

(21) $$\begin{array}{c} \left\{\begin{array}{c} E\left[\phi_{p}\left(x\left[n\right];c_{p}\right)\phi_{j}\left(x\left[n\right];c_{j}\right)\right]=1,p=j\\ E\left[\phi_{p}\left(x\left[n\right];c_{p}\right)\phi_{j}\left(x\left[n\right];c_{j}\right)\right]=0,p\neq j \end{array}\right.. \end{array}$$

For polynomial basis functions with the highest order term of 2p−1(p>1), assuming orthogonality with any basis function of order less than 2p−1, it is possible to derive p−1 orthogonal equations as

(22) $$\begin{array}{c} E\left[\phi_{p}\left(x\left[n\right];c_{p}\right)\phi_{k}\left(x\left[n\right];c_{k}\right)\right]=0,k=1,2,\ldots ,p-1 \end{array}.$$

Furthermore, we can obtain

(23) $$\begin{array}{c} \left\{\begin{array}{c} E\left[\phi_{p}\left(x\left[n\right];c_{p}\right)c_{1,0}x\left[n\right]\right]=0,\left(k=1\right)\\ E\left[\phi_{p}\left(x\left[n\right];c_{p}\right)\left(c_{2,0}x\left[n\right]+c_{2,1}{\left|x\left[n\right]\right|}^{2}x\left[n\right]\right)\right]=0,\left(k=2\right)\\ \;\;\vdots \\ E\left[\phi_{p}\left(x\left[n\right];c_{p}\right)\left(c_{p-1,0}x\left[n\right]+\cdots +c_{p-1,p-2}{\left|x\left[n\right]\right|}^{2p-2}x\left[n\right]\right)\right]=0,\left(k=p-1\right). \end{array}\right. \end{array}$$

Assume $c_{p,p-1}=1,\forall p$. When k=1, we define $\mu_{p}=E\left[{\left(x\left[n\right]\right)}^{p}\right]$; then, (23) can be rewritten as

(24) $$\begin{array}{c} \;\;E\left[\left(c_{p,0}x\left[n\right]+c_{p,1}{\left|x\left[n\right]\right|}^{2}x\left[n\right]+\cdots +{\left|x\left[n\right]\right|}^{2p-2}x\left[n\right]\right)x\left[n\right]\right]\\ =E\left[\left(c_{p,0}{\left|x\left[n\right]\right|}^{2}+c_{p,1}{\left|x\left[n\right]\right|}^{4}+\cdots +{\left|x\left[n\right]\right|}^{2p}\right)\right]\\ =c_{p,0}\mu_{2}+c_{p,1}\mu_{4}+\cdots +\mu_{2p}\\ =0 \end{array},\left(k=1\right).$$

Let $\mu_{2a}^{2b}=\left\{\mu_{2a},\mu_{2a+2},\ldots ,\mu_{2b}\right\},1⩽a⩽b$. Equation (23) can be written in matrix form as

(25) $$C_{p}M_{p}{\bar{c}}_{p}+C_{p}\mu_{2p}^{4p-4}=0,$$

where ${\bar{c}}_{p}$ is a subvector of the $c_{p}={\left[{{\bar{c}}_{p}}^{T}\;\;1\right]}^{T}$ and $C_{p}$ is an invertible lower triangular matrix. The matrix $M_{p}$ is defined as

(26) $$M_{p}=\left[\begin{array}{cccc} \mu_{2} & \mu_{4} & \ldots & \mu_{2p-2}\\ \mu_{4} & \mu_{6} & \ldots & \mu_{2p}\\ \vdots & \vdots & \ddots & \vdots \\ \mu_{2p-2} & \mu_{2p} & \ldots & \mu_{4p-6} \end{array}\right]\mathrm{and}\;\det\left(M_{p}\right)\neq 0$$

Thus, we have

(27) $${\bar{c}}_{p}=-{M_{p}}^{-1}\mu_{2p}^{4p-4}.$$

The instantaneous basis function vector is defined as

(28) $$\Phi \left[n\right]={\left[\phi_{1}\left(x_{L}\left[n\right];c_{1}\right),\ldots ,\phi_{\frac{P+1}{2}}\left(x_{L}\left[n\right];c_{\frac{P+1}{2}}\right)\right]}^{T}.$$

Integrating the nonlinear PA model with the multi-layered joint channel estimator employing the LMS algorithm results in

(29) $$\begin{array}{cc} y\left[n\right] & =\;w^{T}\left[n\right]\Phi \left[n\right]+h^{T}\left[n\right]i\left[n\right]+v\left[n\right]\\ \; & =\left[w^{T}\left[n\right]\;\;h^{T}\left[n\right]\right]\;\;{\left[\Phi^{T}\left[n\right]\;\;i^{T}\left[n\right]\right]}^{T}+v\left[n\right]\\ \; & ={h^{T}}_{joint}\left[n\right]u\left[n\right]+v\left[n\right], \end{array}$$

where $h_{joint}^{T}\left[n\right]=\left[w^{T}\left[n\right]\;\;h^{T}\left[n\right]\right]\;\;$ and $u\left[n\right]={\left[\Phi^{T}\left[n\right]\;\;i^{T}\left[n\right]\right]}^{T}$.

Furthermore, the iterative formula can be expressed as

(30) $${\hat{h}}_{joint}\left[n+1\right]={\hat{h}}_{joint}\left[n\right]+\mu u\left[n\right]e\left[n\right],$$

where μ is the step size.

At the *m*-th layer, the received signal after digital SIC is

(31) $$\begin{array}{c} y_{\left(m+1\right)}\left[n\right]=y_{\left(m\right)}\left[n\right]-{\hat{h}}_{joint\left(m\right)}^{T}\;\;\left[n\right]\Phi \left[n\right]+v\left[n\right]. \end{array}$$

The *M*-layered adaptive nonlinear SIC algorithm is summarized in Algorithm 1 and Table 2.
**Algorithm 1** Adaptive algorithm for nonlinear SIC**Input:** 
received signal y[n], known signals x[n] and i[n], basis order *P*, moment collect length $N_{m}$ and basis function generation interval $N_{int}$**Output:** signal after SIC $\hat{r}\left[n\right]$ and RT channel estimation ${\hat{h}}_{\left(m\right)}^{T}\left[n\right]$     **Initialization:** $n_{s}=1$ and $h_{joint(m)}^{T}\left[n\right]=0$1:**for** n=1,2,…,N **do**2:      **if** $n=n_{s}+N_{m}-1$ **then**3:           $\mu_{p}=\frac{1}{N}\sum_{n_{s}}^{n_{s}+N_{m}-1}{\left(x\left[n\right]\right)}^{p}$4:           **for** $p=2,3,\ldots ,\frac{P+1}{2}$ **do**5:                 ${\bar{c}}_{p}=-{M_{p}}^{-1}\mu_{2p}^{4p-4}$, **basis function** $\phi_{p}\left(x\left[n\right];c_{p}\right)$ **generation**6:           **end for**7:      **end if**8:      **if** $n=n_{s}+N_{int}-1$ **then**9:           $n_{s}=n$10:    **end if**11:    $y_{\left(1\right)}\left[n\right]=y\left[n\right]=h_{joint}^{T}\left[n\right]u\left[n\right]+v\left[n\right]$12:    **for** m=1,2,…,M **do**13:         $e_{\left(m\right)}\left[n\right]=y_{\left(m\right)}\left[n\right]-{\hat{h}}_{joint\left(m\right)}^{T}\left[n\right]u\left[n\right]$14:         ${\hat{h}}_{joint\left(m\right)}^{T}\left[n+1\right]={\hat{h}}_{joint\left(m\right)}^{T}\left[n\right]+\mu u\left[n\right]e_{\left(m\right)}\left[n\right]$15:         ${\hat{w}}_{\left(m\right)}^{T}\left[n+1\right]={\left({\hat{h}}_{joint\left(m\right)}^{T}\left[n+1\right]\right)}_{1:N_{1}}$16:         ${\hat{h}}_{\left(m\right)}^{T}\left[n+1\right]={\left({\hat{h}}_{joint\left(m\right)}^{T}\left[n\right]\right)}_{N_{1}+1:N_{1}+N_{2}}$17:         $y_{\left(m+1\right)}\left[n\right]=y_{\left(m\right)}\left[n\right]-{\hat{w}}_{\left(m\right)}^{T}\left[n\right]x\left[n\right]$18:    **end for**19:    $\hat{r}\left[n\right]=y_{\left(m+1\right)}\left[n\right]$20:**end for**

## 4. Simulation Results

### 4.1. Linear SI Canceler

**Figure 4:**
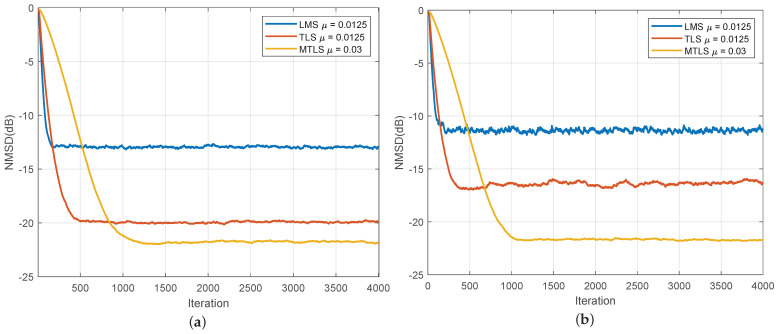
Average NMSD learning curve with different impulsive noise probabilities. (**a**) $P_{i}=0$; (**b**) $P_{i}=0.01$.

In the scenario described by Equation (9), the unknown vector w, with a dimensionality of L=14, conforms to the condition ${∥w∥}^{2}=1$. Additionally, the elements of w are distributed according to a Gaussian distribution with a mean of zero. The input signal is independently generated from zero-mean Gaussian with unit variance. The input and output noises are denoted as $v_{in}=v_{b}$ and $v_{out}=v_{a}+v_{i}$, respectively, where $v_{a}$ and $v_{b}$ are Gaussian-distributed with equal variances of $\sigma_{a}^{2}=\sigma_{b}^{2}=0.1$. The Bernoulli–Gaussian (BG) process is utilized as an impulsive noise model. The impulsive noise $v_{i}\left[n\right]=b\left[n\right]p\left[n\right]$, where b[n] is a Bernoulli process with the probability density function $P\left(b\left[n\right]=1\right)=P_{i},P\left(b\left[n\right]=0\right)=1-P_{i}$ and p[n]∼N(0,10) [26]. Their performance is displayed in Figure 4, quantitatively evaluated via the normalized mean squared difference (NMSD), defined as $=10\log\left(∥{\hat{w}}_{n}{-w∥}^{2}/{∥w∥}^{2}\right)$. It can be observed that the LMS and TLS algorithms decline due to noise interference, whereas the MTLS algorithm remains largely unaffected by such disturbances.

We consider a baseband STAR system characterized by a 5 MHz signal bandwidth. The system operates at a sampling rate of 10 MHz, with both the local and remote transmitters employing binary phase shift keying modulation for signal transmission. The SI and RT channels have lengths specified as $N_{1}=4$ and $N_{2}=10$, respectively. Furthermore, their average energy follows the conditions ISR $=E(∥w{∥}^{2}/∥h{∥}^{2})$ and SNR $=E(∥s\left(n\right){∥}^{2}/\sigma_{a}^{2})$. The received signal y(n) is subject to corruption from two sources: Gaussian white noise $v_{a}$ and random impulsive noise $v_{i}$. The occurrence probability of the impulsive noise is denoted as $P_{i}$. Additionally, the local reference signal i(n) is influenced by Gaussian noise $v_{b}$, where the condition $\sigma_{a}^{2}=\sigma_{b}^{2}$ holds true. In the context of this simulation, we employ the m-MTLS algorithm with a total of number of M=2 layers, which is mainly a compromise considering the additional computational cost brought by multi-layer filters. As *M* increases, the effectiveness of SIC initially improves and then tends to stabilize or even decline. This is due to the propagation of estimation errors across different layers. To assess the efficacy of the proposed multi-layered joint estimator, a comparative analysis is conducted against the minimum mean square error (MMSE) estimator and the single-layer MTLS estimator, focusing on their performance in estimating the RT channel. The physical meaning of the parameters involved in this simulation is summarized in Table 3.

**Figure 5:**
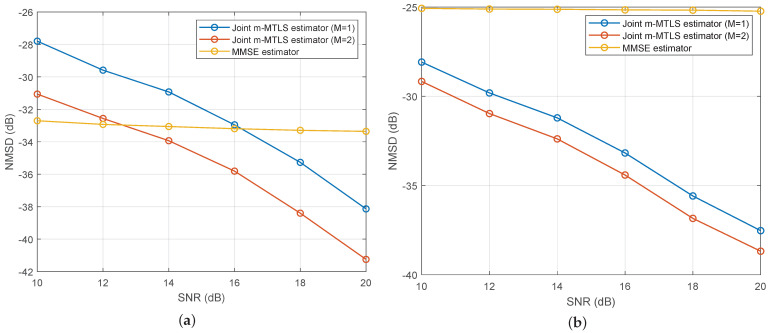
The performance of different estimators under the impulse noise probability. (**a**) $P_{i}=0.01$; (**b**) $P_{i}=0.05$.

Figure 5 illustrates the performance curves of several algorithms in terms of NMSD as the SNR varies across different noise environments, with ISR =20 dB. Compared to the MMSE estimator, the proposed method exhibits higher robustness. The MMSE estimator shows a noticeable decline in performance at certain sampling points when affected by impulsive noise pollution. However, thanks to the M-estimate function, the m-MTLS estimator remains virtually unaffected. Furthermore, as the SNR increases, the NMSD of the m-MTLS estimator gradually decreases, whereas the MMSE estimator experiences limited gain due to convergence being disrupted by interference from the interest signal.

### 4.2. Nonlinear SI Canceler

In this section, We use the Saleh’s PA model [27] to simulate the nonlinear distortion, and the SI signal received after ADC can be described as follows:

(32) $$f\left(x\left[n\right]\right)=\sum_{l=0}^{L-1}h\left[l\right]\frac{\gamma x\left[n-l\right]}{1+\beta {\left|x\left[n-l\right]\right|}^{2}},$$

where γ=3 and β=0.03 in this simulation, and h[n] is the overall memory length, set to L=5.

We assess the performance in terms of the mean squared error (MSE), defined as follows:

(33) $$MSE=E\left[{\left(f\left(x\left[n\right]\right)-\hat{y}\left[n\right]\right)}^{2}\right].$$

**Figure 6:**
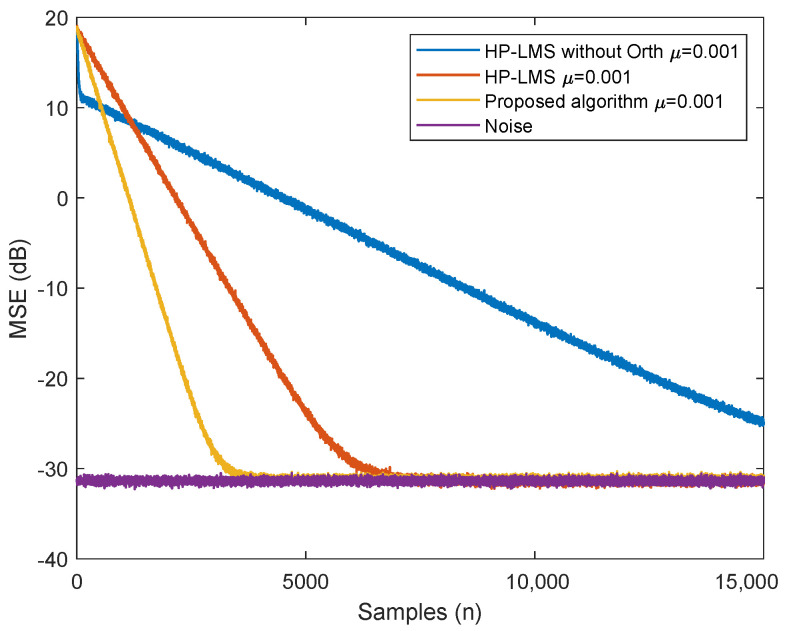
MSE performance of different SIC algorithms with the same step size, when P=5.

The performance of the proposed algorithm and the commonly used LMS algorithms based on HPs are evaluated and the results are shown in Figure 6. In this simulation, we assume the transmitted data follow a uniform distribution in the range [−2.5,2.5] and v[n] is zero-mean Gaussian noise with variance 1.

As shown in Figure 6, compared to existing algorithms based on standard HPs, the proposed algorithm achieves faster convergence while maintaining equivalent MSE performance.

In the nonlinear SIC scenario, we simulate data transmission using a uniform distribution within the range of [−2.5,2.5], and the remote signal i[n]∈{−1,1}. The nonlinear SI canceler is demonstrated in Section 3.2. Considering both computational complexity and algorithm performance, the joint SIC algorithm has a two-layer filtering structure with M=2. The basis functions for all algorithms are of order up to five.

**Figure 7:**
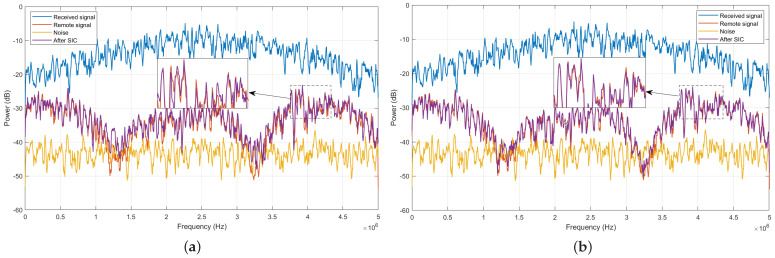
Power spectrum of signals at the receiver, with ISR =20 dB. (**a**) Proposed algorithm; (**b**) HP-LMS algorithm.

**Figure 8:**
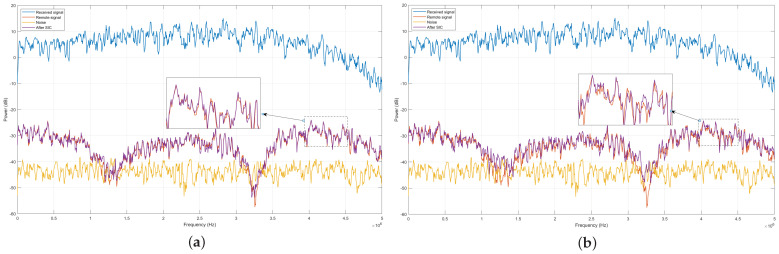
Power spectrum of signals at the receiver, with ISR =40 dB. (**a**) Proposed algorithm; (**b**) HP-LMS algorithm.

Figure 7 and Figure 8 compare the power spectrum of different signals at the receiver for ISR =20 dB and ISR =40 dB, respectively. As seen, in both cases, the signal after SIC more closely approximates the remote signal when using the proposed SIC algorithm compared to the method based on HP-LMS.

**Figure 9:**
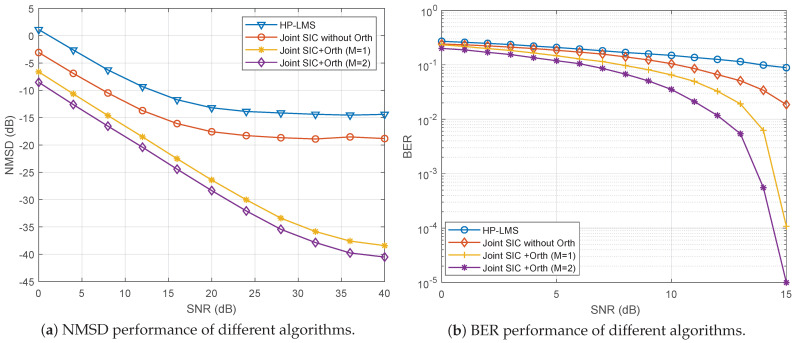
Performance of different algorithms, with ISR =40 dB. (**a**) NMSD performance; (**b**) BER performance.

Figure 9 shows the NMSD and bit error rate (BER) performance of the proposed algorithm and other SIC algorithms. For symbol detection at the receiver, a decision feedback equalizer (DFE) is employed, with both the feedforward and feedback filters having lengths of 15. The DFE employs a truncated version of the transmitted signal as the training signal, with tap weights being trained exclusively during the first block. In this result, as expected, the joint SIC algorithm with a generalized HP basis function with orthogonality exhibits superior performance.

## 5. Conclusions

In this paper, we proposed two adaptive algorithms for linear and nonlinear SIC, based on a multi-layered joint channel estimator structure. In the case of linear SI, given that both the independent and dependent variables are subject to measurement errors, and considering the impact of impulsive noise present in wireless environments on the convergence process of adaptive algorithms, the m-MTLS algorithm is proposed to enhance robustness. For the nonlinear distortion generated by the PA, we combine the joint channel estimator and generalized HP basis function to propose a joint SIC method that outperforms current state-of-the-art SIC algorithms in scenarios of both high and low ISR, as well as high SNR. However, the complexity of this method is higher, mainly due to the increase in the number of basis function coefficients and the length of the filter during joint estimation, as well as the number of layers *M*, which also contributes to the increased complexity.

## Figures and Tables

**Table 1:** Table of acronyms.

Acronym	Definition
STAR	simultaneous transmit and receive
IBFD	in-band full-duplex
SI	self-interference
SIC	self-interference cancellation
RF	Radio Frequency
LNA	low-noise amplifier
PA	power amplifier
LMS	least mean square
PH	parallel Hammerstein
TX	transmitter
RT	remote transmitter
HP	Hammerstein polynomials
SNR	signal-to-noise ratio
ISR	interference-to-signal ratio
m-MTLS	multi-layered M-estimate total least squares
NMSD	normalized mean squared difference
MSE	mean squared error
BER	bit error rate
DFE	decision feedback equalizer

**Table 2:** Table of notations.

Parameter	Symbol
*p*-th order basis functions	$\phi_{p}\left(x\left[n\right];c_{p}\right)$
Coefficients of the *p*-th basis function	$c_{p}$
Nonlinear order of PA	*P*
*p*-th SI channel response	$w_{p}$
*p*-th RT channel response	$h_{p}$
Joint channel response	$h_{joint}$
*p*-th even-order partial moments	$M_{p}$
Number of layers	*M*
Input of *m*-th layers	$y_{\left(m\right)}$

**Table 3:** Table of parameters.

Parameter	Symbol	Value
Length of SI channel	$N_{1}$	4
Length of RT channel	$N_{2}$	10
Layers	*M*	2
Input noise	$v_{in}$	$v_{b}$
Output noise	$v_{out}$	$v_{a}+v_{i}$
Gaussian noise	$v_{a}$, $v_{b}$	$\sigma_{a}^{2}=\sigma_{b}^{2}$
Impulsive noise	$v_{i}$	$\sigma_{i}^{2}=10$
ISR	-	20 dB

## Data Availability

Data are contained within the article.
